# Bremsstrahlung dose of ^165^Dy in radiosynovectomy

**DOI:** 10.1120/jacmp.v15i4.4746

**Published:** 2014-07-08

**Authors:** H.C. Manjunatha

**Affiliations:** ^1^ Department of Physics Government College for Women Kolar Karnataka India

**Keywords:** bremsstrahlung dose, dysprosium‐165, rheumatoid arthritis, radiation synovectomy

## Abstract

There has been an increased interest in ^165^Dy radiossynovectomy, which emits relatively high‐energy (> 1 MeV) beta rays. The production of *in vivo* bremsstrachlung radiation hazards warrants evaluation. The bremsstrahlung component of the decay scheme of ^165^Dy has been traditionally ignored in internal dosimetry calculations. We have estimated the bremsstrahlung dose of ^165^Dy distributed in muscle and bone to body by various internal organs (adrenals, brain, breasts, gallbladder wall, LLI wall, small intestine, stomach, ULI wall, heart wall, kidneys, liver, lungs, muscle, ovaries, pancreas, red marrow, bone surfaces, skin, spleen, testes, thymus, thyroid, urine bladder wall, uterus, fetus, placenta, and total body) during radiosynovictomy. In the present study, muscle and bone are considered to be source organs. These estimated values show that the bremsstrahlung radiation absorbed dose contribution from an organ to itself is very small compared to that originating from the beta source. However, contribution to other organs is not always negligible, especially when large amounts of ^165^Dy may be involved, such as in therapy applications. Hence the component of the total dose due to bremsstrahlung dose should be considered in radiosynovictomy or other therapy applications.

PACS numbers: 34.80.‐i, 78.70.‐g, 33.20.Rm, 34.50.Bw

## I. INTRODUCTION

Rheumatoid arthritis (RA), a systemic disease of the connective tissue, whose alterations occur in the areas of articular, periarticular, and tendinous structures, manifests itself through local inflammation, predominantly in the synovial membrane. Almost half of the patients diagnosed with RA have problems with the knee joint.^(1)^ In the treatment of rheumatoid arthritis, a surgical, chemical, or radiation synovectomy (RSV) may be applied. The first treatment used is the prescription of anti‐inflammatory drugs, steroids, and others.^(2)^ In some cases, this treatment may not be effective, and may cause the formation of pannus and the destruction of the articular cartilage, requiring surgical treatment, arthrodesis, or even total knee replacement. Surgical synovectomy is expensive and may have side effects, such as the possibility of a local infection and loss of joint mobility. Arthroscopic synovectomy of the knee may be less invasive, but the results are limited.^(3)^ When conventional treatment fails or surgery is impossible, radiation synovectomy (RS) may be applied. This technique consists of an intraarticular injection of colloids or macroaggregates bound to radionuclide beta‐emitters. The objective is to destroy the diseased pannus and inflamed synovium by direct and highly selective irradiation, with the expectation that following synovium destruction, the regenerated synovium will be free of disease. It is necessary that the colloidal particle be large enough to remain intraarticular for at least a half‐life of the beta nuclide and avoid irradiation of remote organs. The radiation synovectomy (RS) has the merits of a simple operation and no postoperative complications. In the treatment of arthritis by radiation from radioactive materials in the lesions, the material administered to the lesion should be retained only in the lesion, with no leakage.

Since the introduction of RS in 1952, a large number of radionuclides have been studied, and their usefulness and clinical efficacy were analyzed in several clinical trials. The first beta‐emitting radionuclide for RS was colloidal gold, Au‐198. Though it was clinically effective, it did not gain widespread use because of an unacceptable whole‐body radiation load due to its additional gamma‐emission and a high extraarticular leakage into regional lymph nodes.^(4)^ Thus, as early as 1963, yttrium‐90 was recommended, instead, and is still in use for RS of the knee joint. It is a pure β−emitter with a physical half‐life of 2.7 days and a high energy of 2.26 MeV. Due to its maximum tissue penetration of approximately 11 mm, yttrium‐90 cannot be used to treat joints smaller than the knee because this could lead to damage to articular cartilage or overlying skin.^(5)^ Due to these disadvantages, dysprosium‐165 was recommended as an alternative radionuclide for knee joint RS.

The radionuclides available to RS have a short physical half‐life and emit ionizing particles with an average affective tissue penetration (the maximum penetration is less than 10 mm). The objective is to reach the inflamed synovia, producing an absorbed dose sufficient to eliminate the disease. Another desirable feature is the existence of a combined emission of low‐energy gamma rays that can generate a scintigraphy image to evaluate the quality of the injection and to monitor the migration of radio nuclides in the lymphatic system. It is important that the radioisotope be available, nontoxic, and chemically pure.^(6)^ Thus, dysprosium‐165 (${}^{165}\text{Dy}$) and samarium‐153 (${\;}^{153}\text{Sm}$) present features suitable for use in RS treatment. These radionuclides have a short physical half‐life, emit beta and gamma rays of low energy, and can bind themselves to macroaggregates (particles of an adequate size) so that there is no radioactive spreading in the lymphatic system. Their use is expected to reduce inflammation and pain and improve the articular mobility.^(7)^ Radiation synovectomy (RS) using ${\;}^{165}\text{Dy}$ Ferric‐hydroxide causes no significant radiation burden to most patients as indicated by the absence of adverse changes in levels of biomarkers of cytogenetic damage and a low incidence of leakage.^(8)^

The incorporated/injected ${\;}^{165}\text{Dy}$ during radiosynovectomy produces bremsstrahlung radiation and could have different energies and intensities. The bremsstrahlung yield is a function of two components, namely internal bremsstrahlung and external bremsstrahlung. The intensity of external bremsstrahlung (EB) largely depends on the energy of the emitted beta particles and atomic number of the surrounding matrix material. On the other hand, internal bremsstrahlung component inherently depends on the interaction of the emitted beta particle with the nucleus of the source radionuclide itself. It can, therefore, be stated that the photon characteristics of external bremsstrahlung depend on the surrounding matrix material (tissue), whereas those of internal bremsstrahlung would depend on the emission characteristics of radionuclide. The bremsstrahlung component of beta emitters has been traditionally ignored in internal dosimetry calculations. This may be due to a lack of available methods for including this component in the calculations or to the belief that the contribution of this component is negligible compared to that of other emissions. The phenomenon of bremsstrahlung production is most important at high energies and high medium atomic numbers. In patients with chronic synivitis to rheumatoid arthritis (RA), the results of RSV are favorable. Local instillation of radiopharmaceuticals can reduce effusion.^(9)^ In our previous work,^(10)^ we have formulated a general method to evaluate the EB spectrum and hence the bremsstrahlung dose of therapeutic beta nuclides in bone and muscle only. Radiation therapy needs experimental studies on the exposure due to bremsstrahlung in tissues. But these experiments are very difficult to undertake and analyze, since many biochemical processes are taking place at the same time, competing with radiation effects. The resulting hazard of bremsstrahlung radiation released during beta therapy may, therefore, be some of concern, at least theoretically, and should be systematically evaluated. The injected ${\;}^{165}\text{Dy}$ beta nuclide interacts with surrounding bone and muscle and produce bremsstrahlung radiation. In the present study, we have formulated the method to estimate the radiation dose from the bremsstrahlung component of ${\;}^{165}\text{Dy}$ distributed in the muscle and bone to body for various body organs during radiosynovectomy.

## II. MATERIALS AND METHODS

The computations of bremsstrahlung radiation dose have been divided into three parts, which are as follows:

### A. Estimation of bremsstrahlung cross section

Markowicz et al.^(11)^ proposed an expression for modified atomic number ($Z_{\text{mod}}$) of compound target defined for bremsstrahlung process to take into account the self absorption of bremsstrahlung and electron back scattering:

(1) $$Z_{\text{mod}}=\frac{\sum_{i}^{l}\frac{W_{i}Z_{i}^{2}}{A_{i}}}{\sum_{i}^{l}\frac{W_{i}Z_{i}}{A_{i}}}$$

Here, $W_{i},A_{i}$, and $Z_{i}$ are atomic weight, weight fraction, and atomic number of ith element, respectively. $Z_{\text{modis}}$ is evaluated using Eq. (1) and their composition.^(12)^ The evaluated values of $Z_{\text{mod}}$ for muscle and bone are 6.481 and 10.991, respectively. The bremsstrahlung cross section is evaluated using Lagrange's interpolation technique, Seltzer‐Berger^(13)^ theoretical bremsstrahlung cross section data given for elements using the following expression

(2) $$\sigma_{z_{\text{mod}}}=\sum \left(\frac{\prod_{Z_{\text{mod}}\neq Z}{\text{(z}}_{\text{mod}}-\text{z)}}{\prod_{z\neq Z}\left(z-Z\right)}\right)\sigma_{z}$$

where lower case *z* is the atomic number of the element of known bremsstrahlung cross section $\sigma_{z}$ adjacent to the modified atomic number ($Z_{\text{mod}}$) of the compound whose bremsstrahlung cross section $\sigma_{Z_{\text{mod}}}$ is desired, and upper case *Z* are atomic numbers of other elements of known bremsstrahlung cross section adjacent to $Z_{\text{mod}}$. The estimated $\sigma_{\text{Zmod}}$ (milli barn/MeV) is used for evaluation of spectrum.

### B. Evaluation of Bremsstrahlung spectrum

The number n(T,k) of bremsstrahlung photons of energy k when all of the incident electron energy T completely absorbed in thick target is given by Bethe and Heitler^(14)^ is

(3) $$n(\text{T,k})=N\int_{k}^{T}\left(\frac{\text{σ}(\text{E,k})}{-\text{dE}/\text{dx}}\right)\text{dE}$$

where σ(E,k) is bremsstrahlung cross section at photon energy *k* and electron energy E, *N* is the number of atoms per unit volume of target, and *E* is the energy of an electron available for an interaction with nucleus of the thick target after it undergoes a loss of energy per unit length (dE/dx). For a beta emitter with end point energy $T_{\text{max}}$, spectral distribution of bremsstrahlung photons (S (k)) is given by

(4) $$S(k)=\frac{\int_{T}^{T\max}n(\text{T,k})P(T)\text{dT}}{\int_{T}^{T\max}P(T)\text{dT}}$$

where *P(T)* is the beta spectrum. Evaluated results of σ(E,k) of Eq. (2) and tabulated values of (−dE/dx) of Seltzer‐Berger^(13)^ data are used to get S(k) for the target compounds.

### C. Evaluation of Bremsstrahlung dose

We used the following expression^(15)^ for the calculation of specific absorbed fraction of energy at distance x from the point source monoenergetic photon emitter:

(5) $$\Phi (x)=\frac{\mu_{en}\exp(-\mu x)B_{en}}{4\pi x^{2}\rho }$$

Here $\mu_{en}$ is linear absorption coefficient of photons of given energy, μ *is* linear attenuation coefficient of photons of given energy, $B_{en}$ is energy absorption buildup factor, ρ is density of the medium. The energy absorption buildup factors have been computed using geometric progression (GP) fitting method.^(16)^ The values of $\mu_{\text{en}}$ and $\mu_{\text{of}}$ photons have been taken from Hubbel.^(17)^ The specific absorbed fraction for a given beta source was estimated by integrating over the entire bremsstrahlung spectrum.

(6) $$\Phi (x)=\int_{0}^{T_{\text{max}}}\Phi_{T}(x)dT$$

where $T_{max}$ is the maximum energy of beta. Estimation of the value of Φ allows calculation of the absorbed dose at fixed distances from the point source in the infinite, homogeneous medium

(7) $$D(x)=\tau \sum \Delta_{i}\Phi_{i}(x)$$

where *D(x)* is the absorbed dose at distance x per unit initial activity (Gy/MBq), τ is the residence time of activity, and Δ is the energy emitted per unit cumulated activity and it is numerically equal to (2.13 $n_{i}E_{i}$), where $n_{i}$ is the frequency of occurrence of emissions with energy $E_{i}$; the quantities $n_{i}$ and $E_{i}$ are provided by the calculated bremsstrahlung spectrum using Eq. (4). We have estimated D(x) between x=0.01 and x=10 mm, through complete decay of a ${\;}^{165}\text{Dy}$ beta source. After obtaining the absorbed dose at a number of chosen distances from the source, we have also plotted the calculated estimates of absorbed dose per unit initial activity as a function of distance from the point source. We then developed S‐values^(18)^ for ${\;}^{165}\text{Dy}$ bemsstrahlung emissions for activity uniformly distributed throughout the muscle and cortical bone of the standard reference male phantom^(19)^ by folding the bremsstrahlung spectrum over the specific absorbed fractions^(19)^ as a function of energy for these source regions.

## III. RESULTS & DISCUSSION

**Figure 1:**
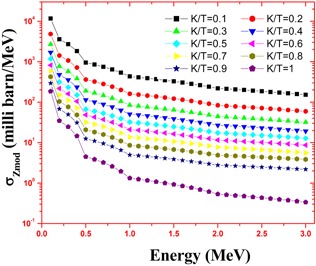
Variation of bremsstrahlung cross section with photon energy in muscle (k and T are photon and electron energies, respectively).

**Figure 2:**
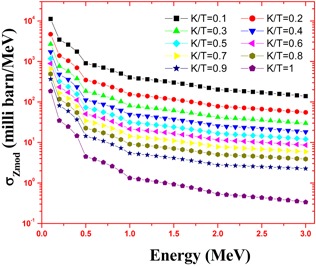
Variation of bremsstrahlung cross section with photon energy in cortical bone (k and T are photon and electron energies, respectively).

**Figure 3:**
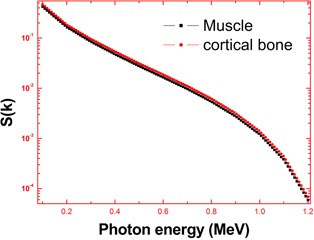
The evaluated bremsstrahlung spectra (S(k)) of ${\;}^{165}\text{Dy}$ expressed as number of photons per $m_{\circ }c^{2}$ per beta.

The calculations employed the accurate energy absorption buildup factors and the bremsstrahlung photon spectrum. This estimated spectrum is accurate because it is based on more accurate $Z_{\text{mod}}$, Seltzer‐Berger data where an electron–electron interaction is also included. The variation of specific absorbed fractions of energy (Φ) with photon energy in cortical bone and muscle are as shown in Figs. 1 and 2. For the purpose of comparison, we have simulated the bremsstrahlung cross sections in bone and muscle using a GEANT4.^(20)^ The cross sections calculated in the present method agrees with that of Geant4 and it is shown in Table 1. The specific absorbed fraction of energy (Φ) increases up to 0.1 MeV and then decreases. The variation of Φ with energy is due to dominance of photoelectric absorption in the lower end and dominance of pair production in the higher photon energy region. During the calculation of Φ values, thickness of penetration depth is considered up to 40 mean free paths. The estimated beta induced bremsstrahlung dose per unit activity (in Gy/MBq) of a ${\;}^{165}\text{Dy}$ point source in a muscle and cartical bone medium, through complete decay is as shown in Fig. 3. We find that the bremsstrahlung dose at about 0.01 mm from ${\;}^{165}\text{Dy}$ source in a muscle would be 1.08 Gy/MBq and that of bone is 23.2 Gy/MBq.

**Figure 4:**
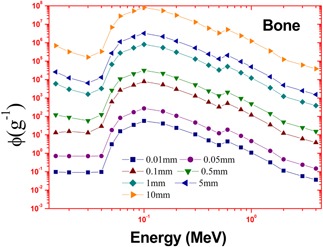
The variation of specific fraction of absorbed energy (Φ) with incident photon energy in bone at penetration depth=40 mfp.

**Figure 5:**
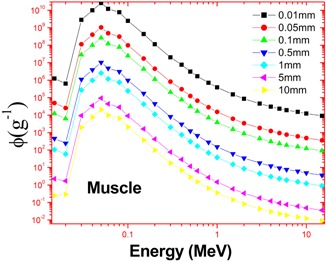
The variation of specific fraction of absorbed energy (Φ) with incident photon energy in muscle at penetration depth=40 mfp.

**Figure 6:**
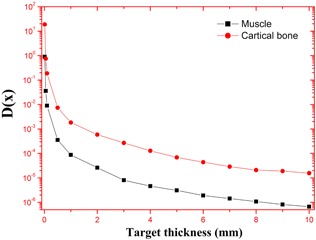
Bremsstrahlunag beta dose per unit activity (in Gy/MBq) of a ${\;}^{165}\text{Dy}$ point source in a muscle and cartical bone medium, through complete decay.

The bremsstrahlung dose of sauraj${\;}^{165}\text{Dy}$ source in a muscle is less than that of cortical bone because it depends on modified atomic number ($Z_{\text{mod}}$) of the target medium ($Z_{\text{mod}}$) of muscle is less than that of cortical bone). In both muscle and bone medium, bremsstrahlung dose decreases with distance (Table 2). Bremsstrahlung dose depends on the specific absorbed fraction of energy (Φ) of the target medium. The variation of specific fraction of absorbed energy (Φ) with incident photon energy in bone and muscle are shown in Figs. 4 and 5. The specific absorbed fraction of energy (Φ) also increases up to the $E_{\text{pe}}$ and then decreases. Here $E_{\text{pe}}$ is the energy value at which the photo electric interaction coefficients match with Compton interaction coefficients for a given material. For a bone and muscle, $E_{\text{pe}}$ is almost equal to 0.1 MeV. The variation of Φ with energy is due to dominance of photoelectric absorption in the lower end and dominance of pair production in the higher photon energy region. In the lower energy end, photoelectric absorption is dominant photon interaction process; hence Φ values are minimum. As the energy of incident photon increases, Compton scattering overtakes the photoelectric absorption. It results in multiple Compton scattering events which increases the value of Φ up to the $E_{\text{pe}}$ and becomes maximum at $E_{\text{pe}}$. Thereafter (above $E_{\text{pe}}$), pair production starts dominating (absorption process), which reduces the value of Φ to minimum. ${\;}^{165}\text{Dy}$ emits relatively high energy (1.286 MeV) beta particles, so that it produces high energy photons (>1.286 MeV), hence dose of ${\;}^{165}\text{Dy}$‐induced bremsstrahlung dose decreases with distance (Fig. 6).

**Table 1:** Comparision of bremsstrahlung cross sections (milli barn/MeV) calculated using present method with that of Geant4

	*Bone*		*Muscle*	
*EnEnergy (MeV)*	*Present Work*	*Using Geant4*	*Present Work*	*Using Geant4*
0.10	185.00	191.00	170.20	176.30
0.50	4.48	5.10	4.13	4.45
1.00	1.32	1.38	1.24	1.25
1.50	0.93	0.97	0.87	0.89
2.00	0.53	0.58	0.50	0.53
2.50	0.43	0.48	0.41	0.41
3.00	0.34	0.39	0.32	0.32

**Table 2:** Bremsstrahlung absorbed dose (in Gy/MBq) near a point source of ${\;}^{165}\text{Dy}$

*Distance (in mm)*	*Source Medium*	
	*Muscle*	*Cortical Bone*
0.01	$1.08\times 10^{+0}$	$2.32\times 10^{+1}$
0.05	$4.31\times 10^{‐2}$	$9.27\times 10^{‐1}$
0.1	$1.08\times 10^{‐2}$	$2.31\times 10^{‐1}$
0.5	$4.25\times 10^{‐4}$	$9.19\times 10^{‐3}$
1	$1.05\times 10^{‐4}$	$2.28\times 10^{‐3}$
5	$3.70\times 10^{‐6}$	$8.50\times 10^{‐5}$
10	$7.96\times 10^{‐7}$	$1.95\times 10^{‐5}$

The estimated S‐values for bremsstrahlung dose (mGy/MBq‐hr) to various target organs from a uniform source of ${\;}^{165}\text{Dy}$ in the muscle and cortical bone are given in Table 3. We have also computed the total equivalent dose due to sum of all other decays of ${\;}^{165}\text{Dy}$ using radiation tool box^(20)^ and compared this with the equivalent dose due to bremsstrahlung radiation (it is given in Table 4). These estimated values show that the bremsstrahlung dose contribution from an organ to itself is very small, but contribution to other organs is not always negligible, especially when large amounts of ${\;}^{165}\text{Dy}$ may be involved as in therapy applications. When using ${\;}^{165}\text{Dy}$ for the radiosynovictomy, the administrated activity varies from 10 to 200 MBq. The estimated component of bremsstrahlung dose compared with overall dose due to injection of ${\;}^{165}\text{Dy}$ beta source is presented in Tables 5 to 7. The component of bremsstrahlung dose is not negligible for higher administrated activities. Bremsstrahlung dose depends on the radionuclide kinetics in a given situation; however, all contributions to total dose should be considered in therapy applications.

**Table 3:** S‐values for bremsstrahlung dose (mGy/MBq‐hr) to various target organs from a uniform source of ${\;}^{165}\text{Dy}$ in the muscle and cortical bone

*Target Organ*	*Muscle*	*Cortical Bone*
Adrenals	$1.119\times 10^{‐5}$	$1.134\times 10^{‐5}$
Brain	$1.451\times 10^{‐7}$	$2.708\times 10^{‐8}$
Breasts	$1.662\times 10^{‐7}$	$2.095\times 10^{‐7}$
Gallbladder Wall	$7.193\times 10^{‐6}$	$1.818\times 10^{‐5}$
LLI Wall	$7.470\times 10^{‐7}$	$1.281\times 10^{‐6}$
Small Intestine	$8.735\times 10^{‐8}$	$1.633\times 10^{‐7}$
Stomach	$2.614\times 10^{‐7}$	$6.740\times 10^{‐7}$
ULI Wall	$3.855\times 10^{‐7}$	$8.003\times 10^{‐7}$
Heart Wall	$3.109\times 10^{‐7}$	$4.697\times 10^{‐7}$
Kidneys	$3.610\times 10^{‐7}$	$5.357\times 10^{‐7}$
Liver	$4.385\times 10^{‐8}$	$6.439\times 10^{‐8}$
Lungs	$1.156\times 10^{‐7}$	$1.496\times 10^{‐7}$
Ovaries	$1.256\times 10^{‐5}$	$2.633\times 10^{‐5}$
Pancreas	$1.188\times 10^{‐6}$	$2.105\times 10^{‐6}$
Red Marrow	$3.039\times 10^{‐7}$	$1.356\times 10^{‐7}$
Bone Surfaces	$1.537\times 10^{‐7}$	$5.816\times 10^{‐8}$
Skin	$2.991\times 10^{‐8}$	$3.316\times 10^{‐8}$
Spleen	$4.640\times 10^{‐7}$	$9.158\times 10^{‐7}$
Testes	$1.860\times 10^{‐6}$	$4.225\times 10^{‐6}$
Thymus	$4.019\times 10^{‐6}$	$8.241\times 10^{‐6}$
Thyroid	$6.420\times 10^{‐6}$	$9.064\times 10^{‐6}$
Urine Bladder Wall	$1.442\times 10^{‐6}$	$4.707\times 10^{‐6}$
Uterus	$1.056\times 10^{‐6}$	$2.890\times 10^{‐6}$

**Table 4:** Comparison of the total equivalent dose (Sv/MBq) due to sum of all other decays of ^165^Dy using radiation tool box^(20)^ with the dose due to bremsstrahlung radiation

	*Overall Dose Due Administrated* ${\;}^{165}Dy$ *Source (from primary and secondary radiation)*	*Bremsstrahlung Dose Due Administrated* ${\;}^{165}Dy$ *Source (from secondary radiations)*	
*Target Organ*	*Muscle*	*Muscle*	*Cortical Bone*
Adrenals	$2.900\times 10^{‐7}$	$1.119\times 10^{‐11}$	$1.134\times 10^{‐11}$
Brain	$2.700\times 10^{‐9}$	$1.451\times 10^{‐13}$	$2.708\times 10^{‐14}$
Breast	$5.500\times 10^{‐7}$	$1.662\times 10^{‐13}$	$2.095\times 10^{‐13}$
LLI	$2.000\times 10^{‐4}$	$7.470\times 10^{‐13}$	$1.281\times 10^{‐12}$
Small Intestine	$4.600\times 10^{‐4}$	$8.735\times 10^{‐14}$	$1.633\times 10^{‐13}$
Stomach	$4.000\times 10^{‐4}$	$2.614\times 10^{‐13}$	$6.740\times 10^{‐13}$
ULI	$5.600\times 10^{‐4}$	$3.855\times 10^{‐13}$	$8.003\times 10^{‐13}$
Kidneys	$5.000\times 10^{‐7}$	$3.610\times 10^{‐13}$	$5.357\times 10^{‐13}$
Liver	$3.300\times 10^{‐7}$	$4.385\times 10^{‐14}$	$6.439\times 10^{‐14}$
Lungs	$9.500\times 10^{‐8}$	$1.156\times 10^{‐13}$	$1.496\times 10^{‐13}$
Ovaries	$1.700\times 10^{‐6}$	$1.256\times 10^{‐11}$	$2.633\times 10^{‐11}$
Pancreas	$9.500\times 10^{‐7}$	$1.188\times 10^{‐12}$	$2.105\times 10^{‐12}$
Red Marrow	$3.500\times 10^{‐7}$	$3.039\times 10^{‐13}$	$1.356\times 10^{‐13}$
Skin	$8.700\times 10^{‐8}$	$2.991\times 10^{‐14}$	$3.316\times 10^{‐14}$
Spleen	$6.000\times 10^{‐7}$	$4.640\times 10^{‐13}$	$9.158\times 10^{‐13}$
Testes	$7.300\times 10^{‐8}$	$1.860\times 10^{‐12}$	$4.225\times 10^{‐12}$
Thymus	$3.700\times 10^{‐8}$	$4.019\times 10^{‐12}$	$8.241\times 10^{‐12}$
Thyroid	$7.000\times 10^{‐9}$	$6.420\times 10^{‐12}$	$9.064\times 10^{‐12}$
Urinary Bladder	$4.000\times 10^{‐7}$	$1.442\times 10^{‐12}$	$4.707\times 10^{‐12}$
Uterus	$1.200\times 10^{‐6}$	$1.056\times 10^{‐12}$	$2.890\times 10^{‐12}$

**Table 5:** Comparison bremsstrahlung dose (Sv) with overall dose due to sum of all other decays of ${\;}^{165}\text{Dy}$ (source organ for all doses is the knee)

	*Estimated Dose for Injection of 10 MBq of* ${\;}^{165}Dy$ *Beta Source*		*Estimated Dose for Injection of 50 MBq of* ${\;}^{165}Dy$ *Beta Source*	
*Organs*	*Overall Dose (Sv)*	*Bremsstrahlung Dose (Sv)*	*Overall Dose (Sv)*	*Bremsstrahlung Dose (Sv)*
Adrenals	$2.900\times 10^{‐6}$	$1.119\times 10^{‐10}$	$1.450\times 10^{‐5}$	$5.595\times 10^{‐10}$
Brain	$2.700\times 10^{‐8}$	$1.451\times 10^{‐12}$	$1.350\times 10^{‐7}$	$7.255\times 10^{‐12}$
Breast	$5.500\times 10^{‐7}$	$1.662\times 10^{‐12}$	$2.750\times 10^{‐6}$	$8.310\times 10^{‐12}$
LLI	$2.000\times 10^{‐3}$	$7.470\times 10^{‐12}$	$1.000\times 10^{‐2}$	$3.735\times 10^{‐11}$
Small Intestine	$4.600\times 10^{‐3}$	$8.735\times 10^{‐13}$	$2.300\times 10^{‐2}$	$4.368\times 10^{‐12}$
Stomach	$4.000\times 10^{‐3}$	$2.614\times 10^{‐12}$	$2.000\times 10^{‐2}$	$1.307\times 10^{‐11}$
ULI	$5.600\times 10^{‐3}$	$3.855\times 10^{‐12}$	$2.800\times 10^{‐2}$	$1.928\times 10^{‐11}$
Kidneys	$5.000\times 10^{‐6}$	$3.610\times 10^{‐12}$	$2.500\times 10^{‐5}$	$1.805\times 10^{‐11}$
Liver	$3.300\times 10^{‐6}$	$4.385\times 10^{‐13}$	$1.650\times 10^{‐5}$	$2.193\times 10^{‐11}$
Lungs	$9.500\times 10^{‐7}$	$1.156\times 10^{‐12}$	$4.750\times 10^{‐6}$	$5.780\times 10^{‐12}$
Ovaries	$1.700\times 10^{‐5}$	$1.256\times 10^{‐10}$	$8.500\times 10^{‐5}$	$6.280\times 10^{‐10}$
Pancreas	$9.500\times 10^{‐6}$	$1.188\times 10^{‐11}$	$4.750\times 10^{‐5}$	$5.940\times 10^{‐11}$
Red Marrow	$3.500\times 10^{‐6}$	$3.039\times 10^{‐12}$	$1.750\times 10^{‐5}$	$1.520\times 10^{‐11}$
Skin	$8.700\times 10^{‐7}$	$2.991\times 10^{‐13}$	$4.350\times 10^{‐6}$	$1.496\times 10^{‐12}$
Spleen	$6.000\times 10^{‐6}$	$4.640\times 10^{‐12}$	$3.000\times 10^{‐5}$	$2.320\times 10^{‐11}$
Testes	$7.300\times 10^{‐7}$	$1.860\times 10^{‐11}$	$3.650\times 10^{‐6}$	$9.300\times 10^{‐11}$
Thymus	$3.700\times 10^{‐7}$	$4.019\times 10^{‐11}$	$1.850\times 10^{‐6}$	$2.010\times 10^{‐10}$
Thyroid	$7.000\times 10^{‐8}$	$6.420\times 10^{‐11}$	$3.500\times 10^{‐7}$	$3.210\times 10^{‐10}$
Urinary Bladder	$4.000\times 10^{‐6}$	$1.442\times 10^{‐11}$	$2.000\times 10^{‐5}$	$7.210\times 10^{‐11}$
Uterus	$1.200\times 10^{‐5}$	$1.056\times 10^{‐11}$	$6.000\times 10^{‐5}$	$5.280\times 10^{‐11}$

**Table 6:** Comparison of bremsstrahlung dose (Sv) to overall dose due to sum of all other decays of ${\;}^{165}\text{Dy}$ (source organ for all doses is the knee)

	*Estimated Dose for Injection of 100MBq of* ${\;}^{165}Dy$ *Beta Source*		*Estimated Dose for Injection of 200MBq of* ${\;}^{165}Dy$ *Beta Source*	
*Organs*	*Overall Dose (Sv)*	*Bremsstrahlung Dose (Sv)*	*Overall Dose (Sv)*	*Bremsstrahlung Dose (Sv)*
Adrenals	$2.900\times 10^{‐5}$	$1.119E\times 10^{-9}$	$5.800\times 10^{‐5}$	$2.238\times 10^{‐9}$
Brain	$2.700\times 10^{‐7}$	$1.451\times 10^{‐11}$	$5.400\times 10^{‐7}$	$2.902\times 10^{‐11}$
Breast	$5.500\times 10^{‐6}$	$1.662\times 10^{‐11}$	$1.100\times 10^{‐5}$	$3.324\times 10^{‐11}$
LLI	$2.000\times 10^{‐2}$	$7.470\times 10^{‐11}$	$4.000\times 10^{‐2}$	$1.494\times 10^{‐10}$
Small Intestine	$4.600\times 10^{‐2}$	$8.735\times 10^{‐12}$	$9.200\times 10^{‐2}$	$1.747\times 10^{‐11}$
Stomach	$4.000\times 10^{‐2}$	$2.614\times 10^{‐11}$	$8.000\times 10^{‐2}$	$5.228\times 10^{‐11}$
ULI	$5.600\times 10^{‐2}$	$3.855\times 10^{‐11}$	$1.120\times 10^{‐1}$	$7.710\times 10^{‐11}$
Kidneys	$5.000\times 10^{‐5}$	$3.610\times 10^{‐11}$	$1.000\times 10^{‐4}$	$7.220\times 10^{‐11}$
Liver	$3.300\times 10^{‐5}$	$4.385\times 10^{‐11}$	$6.600\times 10^{‐5}$	$8.770\times 10^{‐11}$
Lungs	$9.500\times 10^{‐6}$	$1.156\times 10^{‐11}$	$1.900\times 10^{‐5}$	$2.312\times 10^{‐11}$
Ovaries	$1.700\times 10^{‐4}$	$1.256\times 10^{‐9}$	$3.400\times 10^{‐4}$	$2.512\times 10^{‐9}$
Pancreas	$9.500\times 10^{‐5}$	$1.188\times 10^{‐10}$	$1.900\times 10^{‐4}$	$2.376\times 10^{‐10}$
Red Marrow	$3.500\times 10^{‐5}$	$3.039\times 10^{‐11}$	$7.000\times 10^{‐5}$	$6.078\times 10^{‐11}$
Skin	$8.700\times 10^{‐6}$	$2.991\times 10^{‐12}$	$1.740\times 10^{‐5}$	$5.982\times 10^{‐12}$
Spleen	$6.000\times 10^{‐5}$	$4.640\times 10^{‐11}$	$1.200\times 10^{‐4}$	$9.280\times 10^{‐11}$
Testes	$7.300\times 10^{‐6}$	$1.860\times 10^{‐10}$	$1.460\times 10^{‐5}$	$3.720\times 10^{‐10}$
Thymus	$3.700\times 10^{‐6}$	$4.019\times 10^{‐10}$	$7.400\times 10^{‐6}$	$8.038\times 10^{‐10}$
Thyroid	$7.000\times 10^{‐7}$	$6.420\times 10^{‐10}$	$1.400\times 10^{‐6}$	$1.284\times 10^{‐9}$
Urinary Bladder	$4.000\times 10^{‐5}$	$1.442\times 10^{‐10}$	$8.000\times 10^{‐5}$	$2.884\times 10^{‐10}$
Uterus	$1.200\times 10^{‐4}$	$1.056\times 10^{‐10}$	$2.400\times 10^{‐4}$	$2.112\times 10^{‐10}$

**Table 7:** Comparison of bremsstrahlung dose (Sv) to overall dose due to sum of all other decays of ${\;}^{165}\text{Dy}$ (source organ for all doses is the knee)

	*Estimated Dose for Injection of 10000 MBq of* ${\;}^{165}Dy$ *Beta Source*	
*Organs*	*Overall Dose (Sv)*	*Bremsstrahlung Dose (Sv)*
Adrenals	$2.90\times 10^{‐3}$	$3.20\times 10^{‐7}$
Brain	$2.70\times 10^{‐2}$	$4.10\times 10^{‐6}$
Breast	$5.50\times 10^{‐4}$	$3.50\times 10^{‐8}$
LLI	$2.00\times \$\$10^{-0}$	$1.80\times 10^{‐4}$
Small Intestine	$4.60\times 10^{‐0}$	$5.10\times 10^{‐3}$
Stomach	$4.00\times 10^{‐0}$	$3.80\times 10^{‐3}$
ULI	$5.60\times 10^{‐0}$	$5.10\times 10^{‐3}$
Kidneys	$5.00\times 10^{‐3}$	$4.60\times 10^{‐6}$
Liver	$3.30\times 10^{‐3}$	$4.70\times 10^{‐7}$
Lungs	$9.50\times 10^{‐4}$	$9.80\times 10^{‐8}$
Ovaries	$1.70\times 10^{‐2}$	$2.30\times 10^{‐5}$
Pancreas	$9.50\times 10^{‐3}$	$8.20\times 10^{‐7}$
Red Marrow	$3.50\times 10^{‐3}$	$4.10\times 10^{‐4}$
Skin	$8.70\times 10^{‐4}$	$8.10\times 10^{‐7}$
Spleen	$6.00\times 10^{‐3}$	$7.30\times 10^{‐7}$
Testes	$7.30\times 10^{‐4}$	$6.90\times 10^{‐7}$
Thymus	$3.70\times 10^{‐4}$	$1.90\times 10^{‐8}$
Thyroid	$7.00\times 10^{‐5}$	$6.50\times 10^{‐9}$
Uterus	$1.20\times 10^{‐2}$	$2.30\times 10^{‐7}$

## IV. CONCLUSIONS

Calculated S‐values of bremsstrahlung radiation from ${\;}^{165}\text{Dy}$ have been compared with S‐values of beta and all other radiations of same source. These estimated values show that the bremsstrahlung radiation absorbed dose contribution from an organ to itself is very small. But contribution to other organs is not always negligible, especially when large amounts of ${\;}^{165}\text{Dy}$ may be involved as in therapy applications. Hence the component of bremsstrahlung dose to total dose should be considered in radiosynovictomy or other therapy applications.

## ACKNOWLEDGMENTS

The author would like to thank Vision Group on Science and Technology (VGST), Government of Karnataka, India, for providing financial grants in the scheme “Seed Money to Young Scientist Research”.
